# Reciprocal effects of mathematics performance, school engagement and burnout during adolescence

**DOI:** 10.1111/bjep.12548

**Published:** 2022-09-19

**Authors:** Anna Widlund, Heta Tuominen, Johan Korhonen

**Affiliations:** ^1^ Faculty of Education and Welfare Studies Åbo Akademi University Vasa Finland; ^2^ Faculty of Educational Sciences University of Helsinki Helsinki Finland; ^3^ School of Applied Educational Science and Teacher Education University of Eastern Finland Joensuu Finland

**Keywords:** cross‐lagged panel model, longitudinal relations, mathematics performance, school burnout, school engagement

## Abstract

**Background:**

Transitioning into adolescence while simultaneously facing greater academic demands as the level of education increases often entails both academic challenges and general declines in students' school‐related well‐being. Still, however, relatively little is known about the causal relationship between students' academic well‐being (i.e., school engagement and burnout) and their performance during the adolescent years.

**Aims:**

This study examined longitudinal relations between adolescents' mathematics performance, school engagement and burnout (exhaustion, cynicism and inadequacy) across lower secondary education.

**Sample:**

Data came from a longitudinal research project, following Finnish lower secondary school (grades 7–9) students (*N* = 1131) over 4 years (2016–2019).

**Methods:**

Students completed standardized mathematics tests and self‐report measures of school engagement and burnout at four time points, twice within both 7th and 9th grade. A random intercept cross‐lagged panel model (RI‐CLPM) was used to examine pathways between engagement, burnout and mathematics performance over time.

**Results:**

Higher mathematics performance increased students' engagement and lowered their exhaustion and cynicism over time, whereas both engagement and exhaustion predicted higher performance. Negative relations were also found from inadequacy and cynicism on students' mathematics performance. Furthermore, school burnout predicted engagement both positively (from exhaustion) and negatively (from cynicism and inadequacy) within and between the school years, whereas engagement only predicted cynicism and inadequacy negatively within 7th grade.

**Conclusions:**

Findings suggest that the overall relation between students' mathematics performance, engagement and burnout is rather reciprocal, but also, that the relations become more prominent over time, demonstrating the importance of supporting both learning and well‐being in school.

## INTRODUCTION

School engagement and burnout reflect positive and negative, as well as cognitive and affective aspects of students' well‐being, and combined, they are considered central concepts for understanding students' well‐being and adjustment in school (Leiter & Maslach, 2017). Previous findings have demonstrated that positive well‐being often goes together with higher performance (Bücker et al., 2018) but, although the relationship between students' performance, school engagement and burnout is well documented (Lei et al., 2018; Madigan & Curran, 2021), most previous studies have been cross‐sectional, and thus, offer limited evidence in terms of causation.

As the level of education and academic demands increase, many students seem to experience declines in their engagement with schoolwork during the lower secondary school years, whereas school burnout seems to increase (Widlund et al., 2021). Considering also the current (societal) view of mathematics as a key academic domain that has important implications for further educational and occupational success (Korhonen et al., 2014; Widlund et al., 2020), it seems important to extend previous knowledge on how engagement and burnout co‐develop and interrelate with mathematics performance during the critical period of early adolescence. Still, no previous study has investigated the temporal order of such relations (e.g., whether engagement and burnout predict changes in students' performance over time or vice versa), and therefore, this study aimed to complement prior findings by investigating cross‐lagged pathways of adolescents' mathematics performance (test scores), school engagement and burnout (exhaustion, cynicism and inadequacy) across lower secondary education.

### Academic well‐being: School engagement and burnout

The definitions of academic well‐being vary, but many agree that well‐being is a multidimensional phenomenon consisting of both cognitive (what students think of school) and affective (how students feel about school) dimensions (Diener et al., 2018; Putwain et al., 2020). It has often been described as representing subjective evaluations of school, commonly including both positive and negative indicators (Diener et al., 2018; Putwain et al., 2020). Hascher (2008), for example, operationalized academic well‐being as the presence of positive attitudes and enjoyment of school, positive academic self‐concept and the absence of worry, physical complaints, and social problems related to school. Others have, similarly, conceptualized academic well‐being as academic self‐concept, perceived learning difficulties and school burnout (Korhonen et al., 2014), school burnout, engagement, school value and satisfaction with educational choice (Tuominen‐Soini et al., 2012), and school burnout and engagement (Fiorilli et al., 2017).

Consequently, school engagement and burnout were chosen as indicators of academic well‐being in this study, comprising both positive and negative indicators and cognitive and affective components of students' subjective well‐being in school (Diener et al., 2018). Engagement and burnout are commonly combined indicators and key concepts in the job demands–resources theory that has initially been used to explain health and performance in the occupational context (Demerouti et al., 2001). Based on the rationale that school is a place where students work – they attend classes and complete structured activities with specific performance goals – the combined concepts of engagement and burnout may reasonably be extended to educational contexts (Walburg, 2014). The demands–resources theory has increasingly been used in the educational context to explain students' well‐being and academic functioning in school (Study demands–resources theory: Lesener et al., 2020; Salmela‐Aro & Upadyaya, 2014).

School engagement has been described as a positive, study‐related state of mind comprised of energy, dedication and absorption (Salmela‐Aro & Upadyaya, 2012; Schaufeli et al., 2002, 2006). Energy involves high levels of vigour and energy while studying, dedication reflects a positive cognitive attitude towards school, whereas absorption is characterized by being fully concentrated and involved in one's studies. These dimensions are highly correlated (Salmela‐Aro & Upadyaya, 2012) and are commonly described as representing one overall engagement construct (e.g., Salmela‐Aro et al., 2016).

School burnout has typically been approached as a multidimensional construct consisting of three highly correlated, but conceptually distinct factors: exhaustion, cynicism and inadequacy (Salmela‐Aro et al., 2009; Schaufeli et al., 2002). Exhaustion reflects school‐related feelings of strain due to high school demands. Cynicism is described as detached attitudes towards school and loss of interest in one's schoolwork, whereas inadequacy refers to diminished feelings of one's competence and achievements (Salmela‐Aro et al., 2009). These dimensions seem to be differently associated with various school‐related outcomes (e.g., achievement: Salmela‐Aro & Upadyaya, 2014; motivation: Tuominen‐Soini et al., 2012).

Although school engagement and burnout seem to be negatively correlated (Schaufeli et al., 2002), longitudinal studies investigating causality and their reciprocal relations are scarce. However, in line with the assumptions proposed by the demands–resources theory (Bakker & Demerouti, 2017), a meta‐analysis, of which most studies were drawn from occupational contexts, found a reciprocal, negative relationship between engagement and burnout (Maricuțoiu et al., 2017). In the educational context, Salmela‐Aro and Upadyaya (2014) found burnout to negatively predict engagement during one school year in upper secondary education, but not vice versa. Engels et al. (2019), in turn, did not find any relations between behavioural and cognitive components of engagement and school burnout (considered as one factor) during grades 6–9.

### Academic well‐being and performance

School engagement seems to have a positive, moderate association with academic performance, suggesting that highly engaged students generally perform better in school (Lei et al., 2018; Skinner et al., 2008). School burnout, in turn, seems to have negative associations with performance, indicating that students who are exhausted, have cynical attitudes towards school and feel inadequate in school, generally, have lower academic performance (Madigan & Curran, 2021). Such positive relations between engagement and performance, and on the contrary, negative associations between burnout and performance, could be explained, for example, by ‘gain‐ and loss spirals’ as proposed by the job demands–resources theory (Bakker & Demerouti, 2017; Hobfoll et al., 2018). Burnout may develop if the school environment imposes high levels of demands and offers few study resources, whereas the availability of contextual and personal resources fosters school engagement that leads to more positive outcomes (Salmela‐Aro & Upadyaya, 2014). Individuals who are highly engaged in their work or studies likely have more resources to focus and dedicate their energy to their work and may also be more motivated to stay engaged and create their own resources over time, thus creating a ‘gain spiral’ (Bakker & Demerouti, 2017; Hobfoll et al., 2018). However, individuals who experience high strain or burnout are more likely to participate in self‐undermining behaviour, creating more demands over time, thus risking creating a ‘loss spiral’ (e.g., Fiorilli et al., 2017).

Only a few studies have studied the associations between school engagement, burnout and performance longitudinally. Wang et al. (2015) and Paloș et al. (2019) found that prior academic performance (grade point average, GPA) positively predicted school engagement, whereas it negatively predicted school burnout. However, changes in GPA (i.e., the slope factor) were not related to changes in engagement or burnout during Grades 9–11 (Wang et al., 2015). Furthermore, both Paloș et al. (2019) and Salanova et al. (2010) investigated the reversed effect (i.e., of engagement and burnout on performance) among university students. While Salanova et al. (2010) only found engagement to predict subsequent performance, no significant effects were found by Paloș et al. (2019) in that direction.

Parviainen et al. (2020) investigated the longitudinal effects of arithmetic and reading skills on school burnout among slightly younger students and found that lower arithmetic skills in 6th grade indirectly predicted higher levels of exhaustion and cynicism in upper secondary education, mediated by exhaustion and changes in cynicism in lower secondary education. However, surprisingly, they also found that better reading skills in 6th grade predicted higher levels of cynicism in upper secondary education. Engels et al. (2019), on the other hand, did not find any associations between performance, burnout and engagement among students in grades 6–7.

Overall, these findings suggest that the associations between performance, engagement and burnout are not always straightforward. A few studies have also shown that some students seem to be both engaged and burned out by school, while still performing well (Tuominen‐Soini & Salmela‐Aro, 2014; Widlund et al., 2018). Cross‐lagged studies investigating the associations between school burnout and other educational outcomes have found similar results; Salmela‐Aro and Upadyaya (2017) found positive relations between exhaustion, educational aspirations and educational attainment over time.

Furthermore, a relevant factor to consider when investigating the dynamics of students' well‐being and performance seems to be gender. Although there are no notable gender differences in Finnish boys' and girls' mathematics performance (OECD, 2019), girls consistently report higher levels of exhaustion and feelings of inadequacy in school (Finnish Institute for Health and Welfare, 2022; Tuominen‐Soini & Salmela‐Aro, 2014; Widlund et al., 2018). Some have also found girls to be slightly more engaged in their studies (Tuominen‐Soini & Salmela‐Aro, 2014). However, when it comes to developmental trends, Wang et al. (2015) did not find any gender differences in the development of either engagement or burnout during the adolescent years.

## THE PRESENT STUDY

Although performance, engagement and burnout have consistently been found to be correlated among students across various age samples and educational contexts, longitudinal studies investigating causality are both scarce and show rather mixed findings. Of the few longitudinal studies that exist, the majority have focused on older students (i.e., university students: Paloș et al., 2019; Salanova et al., 2010), have used GPA as a measure of students' performance (e.g., Paloș et al., 2019; Salanova et al., 2010) and have not investigated the temporal order of such relations (e.g., whether engagement and burnout are initial predictors of performance or vice versa). Furthermore, commonly used traditional cross‐lagged panel models (CLPM, e.g., Engels et al., 2019; Salanova et al., 2010) have received criticism for not separating between‐person effects from within‐person effects. Therefore, it is unclear whether the findings reflect within‐person effects (i.e., changes within an individual) or, rather, differences between individuals.

Consequently, this study aimed to investigate concurrent and cross‐lagged pathways of adolescents' mathematics performance (test scores), school engagement and burnout (exhaustion, cynicism and inadequacy) during lower secondary education. To address the methodological limitations of previous studies, the present study followed recent recommendations by using random intercept cross‐lagged panel modelling (RI‐CLPM, see Hamaker et al., 2015), thus, separating within‐person variability from between‐person variability. The following research questions were addressed:

1. How are mathematics performance and school engagement related over time?
2. How are mathematics performance and school burnout related over time?
3. How are school engagement and burnout related over time?

Based on previous findings, we expected to find positive relations between performance and engagement (H1) and negative relations between performance and burnout constructs (i.e., exhaustion (H2), cynicism (H3) and inadequacy (H4): Paloș et al., 2019; Wang et al., 2015). We also expected to find negative associations between engagement and exhaustion (H5), cynicism (H6) and inadequacy (H7) over time (Maricuțoiu et al., 2017; Salmela‐Aro & Upadyaya, 2014). However, due to the scarcity and mixed findings of previous studies and considering that these likely have overestimated effects as a result of not separating within‐person and between‐person variability, no specific hypothesis was set regarding the temporal order of such relations.

Lastly, considering that previous findings show some gender differences in the investigated variables, gender was added to our model to account for possible mean level differences between genders. Based on previous findings (Tuominen‐Soini & Salmela‐Aro, 2014; Widlund et al., 2018), we expected that girls might report higher levels of both exhaustion, inadequacy and possibly also engagement.

## METHODS

### Participants and procedure

Data came from a longitudinal project (FRAM: *Adolescents' well‐being and learning in future society*) at Åbo Akademi University, utilizing an accelerated design, following students over 4 years. APA ethical standards were carefully followed in the conduct of the whole project. Participation in the study was voluntary, informed consent forms were collected from the students' parents, and the participants were assured of the confidentiality of their responses. Five public lower secondary schools from different regions of Swedish‐speaking areas of Finland participated in the data collection (Swedish is the second official language in Finland). Overall, Finland is a relatively homogenous country regarding both socioeconomic status and ethnicity (Official Statistics of Finland, 2022).

The participants were recruited in lower secondary school in the fall (T1) and spring (T2) of the school year 2016–2017, when they were in Grade 7 (Cohort 1) and Grade 9 (Cohort 2). The same participants were followed up 2 years later, in the fall (T3) and spring (T4) of the school year 2018–2019, when they were in Grade 9 (Cohort 1) and Grade 11, that is, studying in upper secondary education for the second year (Cohort 2), respectively. However, due to relatively large attrition rates (ca 60%–70%) in Cohort 2 after students transitioned from lower to upper secondary education, partly due to difficulties in locating students, and many deciding to withdraw participation, only data collected in Grades 7 and 9 were included in this study. Consequently, we combined data from Cohort 1 (fall and spring in 7th and 9th grade) and Cohort 2 (fall and spring in 9th grade). Time points for Cohort 2, for which we did not have data, were entered as incomplete data. The missing data patterns were examined with Little's MCAR test, which indicated that the survey data (i.e., engagement and burnout) was missing completely at random in both the first (MCAR) (*χ* ^2^[983] = 988.322, *p* = .446) and second (MCAR) (*χ* ^2^[133] = 154.753, *p* = .095) cohort. The missing data in mathematics performance was missing completely at random in Cohort 2 (MCAR) (*χ* ^2^[4] = 9.287, *p* = .54), but not in Cohort 1 (MCAR) (*χ* ^2^[28] = 59.944, *p* = .000). The full information maximum likelihood approach implemented in Mplus, taking all available information into account when estimating model parameters, was used to deal with missing data (Graham, 2008). All students who participated at least once across the measurement waves were included in this study, resulting in 1131 students (see Table 1).

**TABLE 1:** Sample description by cohort and grade level at each time point of data collection

Time	Cohort 1 (% of total)	Cohort 2 (% of total)
Time 1: Fall 2016		
Grade level	Grade 7	Grade 9
*N*	568 (91%)	473 (93%)
Time 2: Spring 2017		
Grade level	Grade 7	Grade 9
*N*	545 (88%)	444 (87%)
Time 3: Fall 2018		
Grade level	Grade 9	
*N*	450 (72%)	
Time 4: Spring 2019		
Grade level	Grade 9	
*N*	431 (69%)	
Total: T1–T4	622 (100%)	509 (100%)

### Measures

#### Mathematics performance

Mathematics performance was assessed with a standardized online test (KTLT; Räsänen et al., 2013), consisting of adaptive multiple‐choice and open questions on basic arithmetic, applied problem‐solving and algebra. It is intended for Grades 7–9 (13–15 years). The score obtained in the test is based on an item response theory model calculated from a nationally representative sample of students (*M* = 100, *SD* = 15).

#### School engagement

School engagement was measured with the Schoolwork Engagement Inventory (Salmela‐Aro & Upadyaya, 2012). The inventory consists of nine items measuring energy (e.g., *When I study, I feel that I am bursting with energy*), dedication (e.g., *I am enthusiastic about my studies*) and absorption (e.g., *Time flies when I am studying*). The items were assessed through a seven‐point Likert‐type scale ranging from 1 (never) to 7 (every day). A composite score was computed from all items to indicate overall school engagement in this study.

#### School burnout

School burnout was assessed by a nine‐item scale, the School Burnout Inventory (Salmela‐Aro et al., 2009), using a six‐point Likert‐type scale ranging from 1 (*completely disagree*) to 6 (*completely agree*). The inventory consists of three subscales: exhaustion (e.g., *I feel overwhelmed by my schoolwork*), inadequacy (e.g., *I often have feelings of inadequacy in my schoolwork*), and cynicism (e.g., *I feel that I am losing interest in my* schoolwork).

### Data analysis strategy

All analyses were carried out in Mplus (Muthén & Muthén, 1998–2017). Analyses began with examining the structural validity and stability of the measures through confirmatory factor analysis (CFA; see Table S1) as well as establishing measurement invariance across time through longitudinal confirmatory factor analysis (LCFA; see Table S2) and across cohorts through multiple group confirmatory factor analysis (MGCFA; see Table S3). As students' mathematics test score was based on an item response theory model, it was not possible to conduct CFAs on that measure.

Next, a random intercept cross‐lagged panel model (RI‐CLPM) was fitted to the data, following the procedure as described by Hamaker et al. (2015). Autoregressive parameters reflect the extent to which variability in one measure at one time point (e.g., engagement at T4) can be explained by variability in the same measure from the immediately preceding time point (e.g., engagement at T3). The cross‐lagged parameters, in turn, estimate the degree to which variability in one measure at a time point (e.g., engagement at T4) can be explained by variability in a different measure at a preceding time point (e.g., burnout at T3). The standard cross‐lagged panel model (CLPM) approach has received criticism, stating that stable differences between individuals over time cannot be estimated separately from changes in measures within an individual (Hamaker et al., 2015). Therefore, it is challenging to determine the degree to which bidirectional patterns of the longitudinal association between measures can be attributed to changes occurring within students or relative changes between students.

A RI‐CLPM bypasses these challenges by separating within‐person variability from between‐person variability. Consequently, each construct was decomposed into a stable between‐student part and a varying within‐student part. The cross‐lagged effects are thus interpreted as the quantities that express the extent to which the variables influence each other within individuals. To capture stable trait‐like differences between students in performance, school engagement and burnout constructs, five overarching random intercept factors were included, one for each measure. These reflect the trait aspects of performance, school engagement and burnout over time. The four observed scores for each time point were the indicators of each random intercept, with all factor loadings constrained to 1.

The within‐student varying part was captured by regressing each observed score on its own latent factor. The resulting 20 latent factors (i.e., one for each construct per measurement wave) were subsequently used to specify within‐time associations, autoregressive stability paths and cross‐lagged paths. The error variances of the observed scores were constrained to zero, ensuring that all variation in the observed measures was entirely captured by the within‐person and between‐person latent factor structures. Lastly, gender was added to the model as a time‐invariant between‐level predictor.

Due to the complexity of the model, composite scores were used for school engagement and burnout. In all analyses, chi‐square (*χ* ^2^), the comparative fit index (CFI: cut‐off value close to >.95), the Tucker–Lewis Index (TLI: cut‐off value close to >.95), and the root mean square error of approximation (RMSEA; cut‐off value close to <.05) were used as model‐fit indices (Marsh et al., 2004). Descriptive statistics (means and standard deviations) and internal consistencies for each time point and cohort are presented in Table 2 and correlations between all variables in Table 3.

**TABLE 2:** Descriptive statistics for all study variables by time point, gender and cohort

	Cohort 1						Cohort 2					
Variable	*N* (boys/girls)	*M* (boys/girls)	*SD* (boys/girls)	Skew.	Kurt.	α	*N* (boys/girls)	*M* (boys/girls)	*SD* (boys/girls)	Skew.	Kurt.	α
Engagement T1	272/274	4.28/4.23	1.67/1.44	−.34	−.80	.95						
Engagement T2	261/268	4.23/4.01	1.62/1.57	−.20	−.99	.95						
Engagement T3	210/226	3.88/3.90	1.61/1.48	−.05	−.94	.95	212/247	4.00/4.06	1.52/1.44	−.14	−.86	.94
Engagement T4	202/220	3.96/3.90	1.74/1.44	−.11	−.88	.96	207/219	3.89/4.06	1.58/1.57	−.08	−1.01	.96
Exhaustion T1	279/284	2.35/2.92	.98/1.18	.69	.01	.78						
Exhaustion T2	265/273	2.48/3.07	1.13/1.24	.49	−.53	.82						
Exhaustion T3	213/232	2.44/3.24	1.18/1.25	.51	−.48	.86	216/248	2.46/3.22	1.07/1.20	.47	−.42	.83
Exhaustion T4	205/226	2.42/3.23	1.16/1.29	.42	−.61	.86	212/226	2.42/3.09	1.04/1.29	.60	−.32	.83
Inadequacy T1	271/280	2.53/2.95	1.15/1.20	.33	−.64	.56						
Inadequacy T2	267/268	2.70/3.14	1.22/1.39	.28	−.73	.54						
Inadequacy T3	211/234	2.77/3.53	1.33/1.32	.10	−.86	.70	213/251	2.71/3.43	1.17/1.29	.14	−.81	.64
Inadequacy T4	201/227	2.59/3.53	1.29/1.44	.17	−.94	.73	211/229	2.68/3.34	1.36	.19	−.85	.66
Cynicism T1	279/286	2.51/2.55	1.21/1.18	.64	−.11	.77						
Cynicism T2	272/271	2.62/2.64	1.26/1.27	.67	−.14	.79						
Cynicism T3	216/234	2.63/2.80	1.30/1.28	.51	−.53	.84	215/250	2.54/2.75	1.20/1.31	.43	−.57	.80
Cynicism T4	203/227	2.50/2.77	1.29/1.31	.54	−.48	.84	210/228	2.60/2.73	1.20/1.31	.42	−.63	.81
Math T1	262/279	101/99.1	13.1/13.0	−.53	.47							
Math T2	249/257	103/101	16.3/16.3	−.67	.59							
Math T3	231/230	110/108	17.5/15.1	−.44	.49		209/196	109/109	17.2/14.1	−.42	.98	
Math T4	219/240	111/109	17.7/15.4	−.57	.40		196/217	111/112	18.9/15.2	−.41	.48	

Abbreviations: Kurt = kurtosis; Math = mathematics performance; Skew = skewness.

**TABLE 3:** Correlations between all study variables by time point and cohort

Variable		1	2	3	4	5	6	97	8	9	10	11	12	13	24	25	26	27	28	29	30
1	Engagement T1	1																			
2	Engagement T2	.66	1																		
3	Engagement T3	.53	.61	1	.69			−.13	−.16			−.26	−.25			−.42	−.36			.21	.21
4	Engagement T4	.53	.60	.74	1			−.17	−.22			−.29	−.34			−.39	−.48			.19	.25
5	Exhaustion T1	−.25	−.23	−.18	−.21	1															
6	Exhaustion T2	−.14	−.20	−.13	−.10	.60	1														
7	Exhaustion T3	−.11	−.13	−.18	−.11	.48	.56	1	.65			.69	.53			.59	.40			−.09	−.06
8	Exhaustion T4	−.03	−.07	−.10	−.08	.42	.51	.71	1			.49	.71			.39	.59			−.11	−.17
9	Inadequacy T1	−.35	−.39	−.27	−.30	.59	.56	.34	.33	1											
10	Inadequacy T2	−.29	−.37	−.31	−.28	.49	.68	.40	.37	.83	1										
11	Inadequacy T3	−.22	−.24	−.36	−.30	.43	.46	.72	.53	.44	.47	1	.61			.63	.43			−.17	−.15
12	Inadequacy T4	−.09	−.16	−.22	−.23	.41	.41	.59	.74	.42	.43	.63	1			.47	.66			−.18	−.21
13	Cynicism T1	−.50	−.42	−.36	−.38	.52	.27	.26	.24	.49	.40	.35	.27	1							
24	Cynicism T2	−.44	−.54	−.43	−.36	.38	.57	.29	.28	.56	.63	.40	.31	.59	1						
25	Cynicism T3	−.35	−.36	−.54	−.44	.32	.31	.55	.39	.36	.38	.69	.44	.49	.51	1	.59			−.10	−.10
26	Cynicism T4	−.16	−.23	−.34	−.41	.32	.28	.42	.61	.32	.33	.45	.65	.38	.41	.60	1			−.15	−.21
27	Math T1	.17	.13	.11	.14	−.10	−.10	−.11	−.09	−.17	−.16	−.10	−.08	−.20	−.15	−.17	−.08	1			
28	Math T2	.23	.23	.19	.20	−.23	−.24	−.22	−.12	−.21	−.22	−.15	−.14	−.24	−.31	−.24	−.16	.63	1		
29	Math T3	.27	.25	.31	.31	−.17	−.06	−.10	−.08	−.20	−.19	−.15	−.13	−.29	−.24	−.24	−.19	.67	.68	1	.71
30	Math T4	.20	.24	.31	.35	−.17	−.13	−.16	−.15	−.18	−.22	−.19	−.16	−.30	−.31	−.27	−.22	.65	.68	.80	1

*Note*: Values below the diagonal refer to students from Cohort 1 and values above the diagonal from Cohort 2.

## RESULTS

### Cross lagged pathways

The RI‐CLPM fitted the data very well [χ^2^(75) = 102.781, *p* = .0183, RMSEA = .018, CFI = .997, TLI = .991] and explained 27% of the variance in the latent mathematics performance variable, 33% in engagement, 23% in exhaustion, 18% in cynicism and 20% of the variance in inadequacy at T4 (i.e., end of 9th grade). The effect of gender was significant on the random intercepts of exhaustion and inadequacy, suggesting that girls felt more exhausted and inadequate in school in general, compared to boys. The between‐student correlations indicated that students with higher mathematics performance were also, generally, more engaged (*r* = .23, *p* < .05), less exhausted (*r* = −.22, *p* < .05), less cynical (*r* = −.29, *p* < .05) and felt less inadequate in school (*r* = −.27, *p* < .05) across measurement waves. The between‐student correlations between engagement and burnout constructs were also significant and negative (exhaustion: *r* = −.32, *p* < .05; cynicism: *r* = −.51, *p* < .05; inadequacy: *r* = −.42, *p* < .05).

**FIGURE 1:**
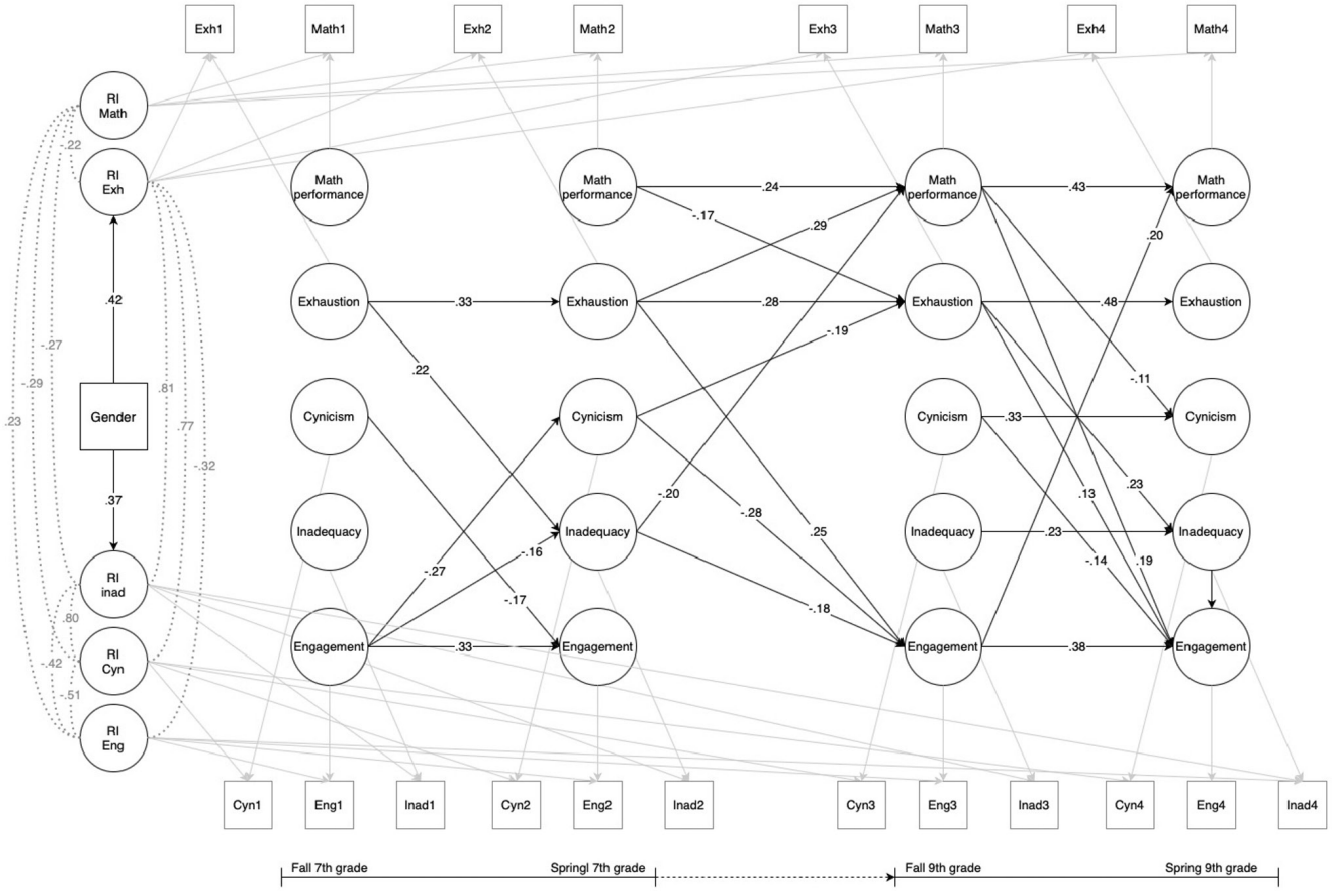
Random intercept cross‐lagged panel model for mathematics performance, school engagement, exhaustion, cynicism, and inadequacy.

Standardized results are depicted in Figure 1.

#### Relations between mathematics performance and school engagement

No significant cross‐lagged effects were detected between mathematics performance and school engagement within 7th grade (T1–T2), or between 7th and 9th grade (T2–T3), suggesting that a student's performance (or engagement) at measurement waves 2 and 3, did not depend on that student's level of engagement (or performance) at the immediately preceding measurement wave. However, there was a reciprocal relationship between performance and engagement within the last year of lower secondary education, indicating that students with higher mathematics performance (relative to the student's own mean) at the beginning of 9th grade were likely relatively more engaged in school later within the same school year. Similarly, students who were more engaged at the beginning of 9th grade likely performed higher in mathematics at the end of the school year.

#### Relations between mathematics performance and school burnout

No significant pathways were detected between performance and burnout within 7th grade. However, students who performed lower in mathematics at the end of 7th grade were likely relatively more exhausted at the beginning of 9th grade. The cross‐lagged path from exhaustion to performance was also significant, but the effect was positive, indicating that higher levels of exhaustion in 7th grade, predicted higher performance in 9th grade. Furthermore, a negative effect was also found from inadequacy in 7th grade on mathematics performance in 9th grade. In 9th grade, higher mathematics performance lowered students' cynical attitudes towards school within the school year.

#### Relations between school engagement and burnout

Both exhaustion and cynicism predicted school engagement between all measurement waves, but the effects differed. Students with elevated levels of exhaustion at each measurement wave were likely to be more engaged in their schoolwork, both within the school years and long‐term, from 7th to 9th grade. The effect of cynicism, however, was negative, indicating that cynical attitudes lowered students' engagement over time. Feelings of inadequacy also negatively predicted students' engagement, but only long‐term.

Engagement only had significant effects on school burnout within the first year of lower secondary education, as initial high levels of engagement lowered students' feelings of inadequacy and cynicism within 7th grade.

## DISCUSSION

While investigating the longitudinal relationships between mathematics performance, school engagement and burnout (exhaustion, cynicism and inadequacy) across lower secondary education, we identified both short‐term (i.e., within grades) and long‐term (i.e., from 7th to 9th grade) relations. Overall, the relationship between all investigated variables seemed to be rather reciprocal, as mathematics performance, school engagement and burnout predicted each other at least once across the measurement waves.

First, as expected (H1), students who performed well in mathematics, also became more engaged with their schoolwork over time (Engels et al., 2019; Paloș et al., 2019), whereas high engagement also predicted higher performance (Salanova et al., 2010). These reciprocal findings fit well within the assumptions proposed by the demands–resources theory (Bakker & Demerouti, 2017; Hobfoll et al., 2018), suggesting that engaged students may have more resources to focus and dedicate their time and energy to their schoolwork, leading to continued high engagement and performance, creating a ‘gain spiral’ over time. However, these relations were only found within the last year of lower secondary school. Thus, it might be that the relation between students' positive feelings and views about school and their performance becomes more salient over time.

Second, confirming our hypotheses (H2, H3 and H4), reciprocal relationships were also found between mathematics performance and burnout (Madigan & Curran, 2021), although the predictions between the distinct burnout constructs varied. A reciprocal relationship was found between performance and exhaustion, but the effects they had on each other differed. In line with previous findings (Paloș et al., 2019; Parviainen et al., 2020), higher performance in 7th grade lowered students' exhaustion over the course of lower secondary education. However, the reversed effect of exhaustion on mathematics performance was positive, possibly reflecting findings of previous person‐centred studies, where groups of students have been identified that both perform well in school, are highly motivated and experience elevated levels of exhaustion (Tuominen‐Soini & Salmela‐Aro, 2014; Widlund et al., 2018). These groups have often been overrepresented by girls, and in the present study, gender also had a significant effect on both the engagement and exhaustion between‐student factors, suggesting that girls were generally more engaged and exhausted in school than boys. Positive effects have also previously been found between, for example, exhaustion and students' educational aspirations and attainment (Salmela‐Aro & Upadyaya, 2017; Widlund et al., 2020). Taken together, this suggests that exhaustion in school might not always have completely negative consequences for students' educational outcomes. Some may feel exhausted by school, potentially as a cost of having high school values and ambitious aspirations and might, therefore, result in higher performance. However, although exhaustion may contribute to higher performance for some at the beginning of adolescence, this association dissipated, while the within‐person correlation between exhaustion and performance in 9th grade turned negative. Over time, prolonged feelings of exhaustion may stop functioning as a positive predictor and have negative consequences for students' performance later on. Consequently, despite these positive effects, students' elevated levels of exhaustion in school should be taken seriously, particularly as it may develop into more serious mental health problems (Fiorilli et al., 2017; Salmela‐Aro et al., 2009), if prolonged.

The results also suggested that students who felt inadequate in school in 7th grade, likely performed lower in mathematics later, in 9th grade. As no previous longitudinal studies exist on the relation between inadequacy and performance, the results clarify previous cross‐sectional findings regarding the temporal order of such associations, suggesting that students' negative feelings towards themselves as students might be an initial predictor of their performance, rather than vice versa. In fact, mathematics performance did not predict inadequacy at any time point in this study. This finding highlights the importance of supporting adolescents' feelings of themselves as students early on, as it may have long‐term negative effects on their performance.

Furthermore, as expected (H3: Paloș et al., 2019; Parviainen et al., 2020; Wang et al., 2015) a negative relation was detected between mathematics performance and cynicism, as higher performance lowered students' cynical attitudes towards school within 9th grade. Overall, these findings may reflect ‘gain spirals’ as proposed by the demands–resources theory (Bakker & Demerouti, 2017), suggesting that individuals who perform well in school likely have better resources to cope with study‐related demands and even create more resources for themselves over time, resulting in lowered feelings of cynicism.

Lastly, the effects of school burnout on students' engagement were, generally, somewhat more prominent than the reversed effects (i.e., the effects of engagement on burnout), as higher engagement only lowered students' feelings of cynicism and inadequacy in 7th grade, but not over the following years. Similarly, to the effects found from exhaustion on students' performance, and other educational outcomes (i.e., aspirations: Salmela‐Aro & Upadyaya, 2017), students' feelings of exhaustion consistently increased their engagement in school, whereas cynicism (short‐ and long‐term) and inadequacy (long‐term), as expected (H6, H7), lowered students' engagement over time.

### Limitations and future directions

Due to the complexity of the model, sum scores were used instead of latent factors (engagement and burnout constructs) in the analyses. Consequently, not all measurement errors are parcelled out in the measurement models, which might underestimate relations between variables. We also found that the Cronbach's alphas for inadequacy were rather low in 7th grade, indicating that the items might not be as adequately representative of inadequacy among younger students, or that more items may be needed to fully capture students' feelings of inadequacy. Furthermore, although combining cohort data comes with several benefits (e.g., increased sample size and time efficiency), it also creates more uncertainty in the data due to increased missing values. Although most data were found to be missing completely at random, math performance in the first cohort was not, which should also be acknowledged when interpreting the results.

Our findings revealed that the relationships between academic well‐being and performance seemed to become more prominent over time during adolescence, and therefore, it would be important for future studies to invest such relations among older students as well, particularly over the educational transition to upper secondary education as this has been found to spark changes in students' well‐being and motivation. It should also be acknowledged that only mathematics performance was used to represent academic performance in this study. Thus, it would be important for future research to investigate the relationship between academic well‐being with performance in other domains as well (e.g., language arts or science), and, to use domain‐specific aspects of students' well‐being in school (e.g., domain‐specific emotional cost).

Lastly, research indicates that the role of demands and resources for an individual's engagement and perceived burnout might be affected by societal cultural beliefs and values (Rattrie et al., 2020). Therefore, the country‐specific context should be acknowledged when making conclusions about our findings, and the relation between students' performance, engagement, and burnout should be tested in different cultures in future work.

### Practical implications

The findings demonstrate the importance of students' emotional engagement with schoolwork, but also, the importance of acknowledging possible strains students' negative feelings towards school might have on their performance. The results indicate that experiences of school burnout, particularly feelings of inadequacy and cynical attitudes, may affect students' performance negatively over time. Therefore, both academic performance and well‐being should be supported in school. It is also important to emphasize that even though exhaustion may go hand in hand with higher performance for some, prolonged feelings of strain may result in more negative outcomes later on. Considering that there were negative effects of school burnout, not only long‐term but also within school years, it would be important to implement resources to support students' academic well‐being early on. Schools should, for example, make student welfare services (i.e., school psychologists and health care) easily available and focus on enhancing adequate coping strategies for students, to help them prevent and handle possible feelings of burnout (van Loon et al., 2020).

## CONCLUSION

This study demonstrates that there seems to be a reciprocal relationship between mathematics performance, engagement and burnout during adolescence. However, while reciprocal relationships were detected between engagement and burnout already at the beginning of adolescence, the relations between students' mathematics performance and academic well‐being seemed to become more prominent during the later years of lower secondary education. The findings also demonstrate the importance of studying the sub‐dimensions of burnout separately, as these seem to be differently related to students' performance, demonstrating the multidimensional nature of school burnout. Overall, the results highlight the importance of supporting, not only students' performance but their well‐being in school as students' views and feelings towards school are clearly affecting their learning processes in mathematics.

## AUTHOR CONTRIBUTIONS

**Anna Widlund:** Conceptualization; formal analysis; writing – original draft. **Heta Tuominen:** Conceptualization; writing – review and editing. **Johan Korhonen:** Conceptualization; project administration; writing – review and editing.

## CONFLICT OF INTEREST

All authors declare no conflict of interest.

## Supporting information


Tables S1–S3

## Data Availability

The data that support the findings of this study are available from the corresponding author upon reasonable request.
