# Supplementary material for: Reciprocal effects of mathematics performance, school engagement and burnout during adolescence
Source: Br J Educ Psychol. 2022 Sep 19;93(1):183–97. doi: 10.1111/bjep.12548 (PMC10087934; doi:10.1111/bjep.12548)
Supplement: Supplementary file 1 — Tables S1–S3 [file BJEP-93-183-s001.docx]

**Supporting Information for**

**Reciprocal Effects of Mathematics Performance, School Engagement, and Burnout**

**During Adolescence**

**Table S1**

*Nested Model Comparisons (1 vs 3 factor solutions) for School Engagement and School Burnout at Time-Points 1–4.*

| Measure | Time point | | Factor(s) | *N* | χ^2^ | df | CFI | TLI | RMSEA | *p* |
| --- | --- | --- | --- | --- | --- | --- | --- | --- | --- | --- |
| School engagement | 1 | | 1 | 585 | 158.231 | 27 | .984 | .930 | .091 | .000 |
|  |  |  | 3 | 585 | 141.851 | 24 | .953 | .930 | .092 | .000 |
|  | 2 | | 1 | 554 | 218.427 | 27 | .926 | .902 | .113 | .000 |
|  |  |  | 3 | 554 | 192.199 | 24 | .935 | .903 | .112 | .000 |
|  | 3 |  |  |  |  |  |  |  |  |  |
|  |  | Cohort 1 | 1 | 454 | 208.220 | 27 | .921 | .895 | .122 | .000 |
|  |  |  | 3 | 454 | 168.515 | 24 | .937 | .906 | .115 | .000 |
|  |  | Cohort 2 | 1 | 476 | 178.690 | 27 | .921 | .895 | .109 | .000 |
|  |  |  | 3 | 476 | 138.881 | 24 | .940 | .910 | .100 | .000 |
|  | 4 |  |  |  |  |  |  |  |  |  |
|  |  | Cohort 1 | 1 | 436 | 171.446 | 27 | .935 | .914 | .111 | .000 |
|  |  |  | 3 | 436 | 142.301 | 24 | .947 | .921 | .106 | .000 |
|  |  | Cohort 2 | 1 | 443 | 140.256 | 27 | .948 | .931 | .097 | .000 |
|  |  |  | 3 | 443 | 122.586 | 24 | .955 | .932 | .096 | .000 |
| School burnout | 1 | | 1 | 578 | 203.327 | 27 | .849 | .799 | .106 | .000 |
|  |  |  | 3 | 578 | 82.338 | 24 | .950 | .925 | .065 | .000 |
|  | 2 | | 1 | 551 | 203.899 | 27 | .866 | .821 | .112 | .000 |
|  |  |  | 3 | 551 | 97.910 | 24 | .947 | .921 | .075 | .000 |
|  | 3 |  |  |  |  |  |  |  |  |  |
|  |  | Cohort 1 | 1 | 452 | 288.321 | 27 | .831 | .774 | .146 | .000 |
|  |  |  | 3 | 452 | 87.736 | 24 | .959 | .938 | .077 | .000 |
|  |  | Cohort 2 | 1 | 475 | 150.982 | 27 | .904 | .871 | .098 | .000 |
|  |  |  | 3 | 475 | 46.508 | 24 | .982 | .974 | .044 | .000 |
|  | 4 |  |  |  |  |  |  |  |  |  |
|  |  | Cohort 1 | 1 | 436 | 237.572 | 27 | .853 | .804 | .134 | .000 |
|  |  |  | 3 | 436 | 69.174 | 24 | .968 | .953 | .066 | .000 |
|  |  | Cohort 2 | 1 | 442 | 182.595 | 27 | .977 | .836 | .114 | .000 |
|  |  |  | 3 | 442 | 65.518 | 24 | .967 | .951 | .063 | .000 |

**Table S2**

*The Goodness of Fit Statistics for Alternative Models Testing Longitudinal CFA*

| Model | | χ^2^ | *df* | CFI | TLI | RMSEA | ΔCFA | ΔRMSEA | *p* |
| --- | --- | --- | --- | --- | --- | --- | --- | --- | --- |
| Cohort 1 |  |  |  |  |  |  |  |  |  |
| *N* = 617 | Configural invariance | 3903.984 | 2256 | .935 | .927 | .034 |  |  | .000 |
|  | Factorial invariance | 3980.226 | 2298 | .934 | .927 | .034 | .001 | .000 | .000 |
|  | Scalar invariance | 4352.318 | 2353 | .922 | .915 | .037 | .012 | .003 | .000 |
| Cohort 2 |  |  |  |  |  |  |  |  |  |
| *N* = 505 | Configural invariance | 1218.907 | 548 | .933 | .923 | .049 |  |  | .000 |
|  | Factorial invariance | 1233.209 | 562 | .933 | .925 | .049 | .000 | .000 | .000 |
|  | Scalar invariance | 1322.984 | 581 | .926 | .919 | .050 | .007 | .001 | .000 |

*Note*. Measurement invariance was tested for T1–T4 for Cohort 1, and T3–T4 for Cohort 2.

**Table S3**

*The Goodness of Fit Statistics for Alternative Models Testing Multiple Group CFA between Cohorts*

| Model | | χ^2^ | *df* | CFI | TLI | RMSEA | ΔCFA | ΔRMSEA | *p* |
| --- | --- | --- | --- | --- | --- | --- | --- | --- | --- |
| T3 |  |  |  |  |  |  |  |  |  |
| *N* = 930 | Configural invariance | 1168.137 | 258 | .921 | .906 | .087 |  |  | .000 |
|  | Factorial invariance | 1173.329 | 272 | .921 | .912 | .084 | .000 | .003 | .000 |
|  | Scalar invariance | 1208.653 | 286 | .919 | .915 | .083 | .002 | .001 | .000 |
| T4 |  |  |  |  |  |  |  |  |  |
| *N* = 882 | Configural invariance | 1039.446 | 258 | .936 | .925 | .083 |  |  | .000 |
|  | Factorial invariance | 1044.935 | 272 | .937 | .929 | .080 | .001 | .003 | .000 |
|  | Scalar invariance | 1077.768 | 286 | .936 | .931 | .079 | .001 | .001 | .000 |
